# Exploration of New Electroacupuncture Needle Material

**DOI:** 10.1155/2012/612545

**Published:** 2012-05-20

**Authors:** Sanghun Lee, Gwang-Ho Choi, Chang Hoon Lee, Yu Kyoung Kim, Saebhom Lee, Sungjin Cho, Sunhee Yeon, Sun-Mi Choi, Yeon-Hee Ryu

**Affiliations:** ^1^Acupuncture, Moxibustion & Meridian Research Center, Korea Institute of Oriental Medicine, Daejeon 305-811, Republic of Korea; ^2^Technical Research Center, Dong Bang Acupuncture, Inc., Boryeong 355-851, Republic of Korea

## Abstract

*Background*. Electro Acupuncture (EA) uses the acupuncture needle as an electrode to apply low-frequency stimulation. For its safe operation, it is essential to prevent any corrosion of the acupuncture needle. *Objective*. The aim of this study is to find an available material and determine the possibility of producing a standard EA needle that is biocompatible. *Methods*. Biocompatibility was tested by an MTT assay and cytotoxicity testing. Corrosion was observed with a scanning electron microscope (SEM) after 0.5 mA, 60 min stimulation. The straightness was measured using a gap length of 100 mm, and tensile testing was performed by imposing a maximum tensile load. *Results*. Phosphor bronze, Ni coated SS304, were deemed inappropriate materials because of mild-to-moderate cytotoxicity and corrosion. Ti-6Al-4V and SS316 showed no cytotoxicity or corrosion. Ti-6Al-4V has a 70 times higher cost and 2.5 times lower conductivity than SS316. The results of both straightness and tensile testing confirmed that SS316 can be manufactured as a standard product. *Conclusion*. As a result, we confirmed that SS316 can be used a new EA electrode material. We hope that a further study of the maximum capacity of low-frequency stimulation using an SS316 for safe operation.

## 1. Introduction

Electroacupuncture is a combination of acupuncture from oriental medicine and low-frequency (1–1,000 Hz) stimulation, which is a type of physical therapy used in western medicine. Low-frequency stimulation was first proposed in 1816 by Louis Berlioz of France, who suggested that electrical stimulation combined with acupuncture treatment could enhance the effectiveness of the treatment. Later, in 1825, Sarlandiere used this technique to treat gout and neurological diseases, and he published a report in which he referred to the technique as “galvanopuncture,” from which the term electroacupuncture is derived [1]. Since that time, electroacupuncture has been used by many researchers who have noted a variety of effects, such as an increased pain threshold [2], increased gastrointestinal movement [3], and weight loss [4], and as a result, there has been an increase in the technique's application in clinics worldwide. However, due to the electrical properties of the current generated by a low-frequency stimulator, electro acupuncture poses safety problems that are distinct from those of traditional acupuncture. Lytle et al. [5] have identified a number of elements related to the safety of the electrical properties of the current generated by a low-frequency stimulator, such as voltage, waveforms, and the pulse frequency and width. Cummings [6] has also focused upon a number of safety elements, such as the possibility of a change in needle depth during electrical stimulation, risk of damage to internal organs due to muscle contraction and needle vibration, needle corrosion due to excessive charging, the caution required when treating areas at risk of shock, such as the area around carotid artery, as well as concerns regarding the interaction of the instrument with patients wearing pacemakers. In particular, Park [7] and Jin [8] studied several electro acupuncture devices and reported that there was no significant difference in corrosion, hardness, or cytotoxicity on the needle surface and tip before and after its operation, while Hwang [9] reported that corrosion was observed in the interface between skin and air when the current is above 0.05 mA and that this corrosion increased with an increase in time or in the applied current. Of the austenitic stainless steels, SS 304 is reported to be most vulnerable to corrosion [10]. It becomes vulnerable to corrosion due to sensitization from thermal treatment, as Cr is extracted at 400~800°C, and the Cr-exhausted region causes corrosion [11]. In the investigation of the corrosion safety of SS 304 ear-acupuncture needles, the authors have confirmed that defective processing of the needle point and surface leads to more severe corrosion [12], a finding that is considered applicable to needles that undergo a similar manufacturing process. In the present study, 4 materials—2 materials with higher conductivity and 2 materials with a higher safety than current material—were tested for the possibility of being used as new material for an electroacupuncture needle. The most appropriate material was selected, and its specifications were evaluated to replace the current new acupuncture needles with a new material.

## 2. Materials and Methods

### 2.1. Experimental Materials

Among the metal wires that are commercially available, we selected phosphor bronze (hereafter referred to as PB) and Ni-coated SS 304 (hereafter referred to as SS 304 Ni), which both have superior electrical conductivity as compared to SS 304, which is currently used for electro acupuncture, along with titanium alloy (Ti-6-Al-4V) and SS 316, which have relatively low electrical conductivity but were expected to demonstrate superior stability. Due to the circumstances involved in purchasing, a thickness of 0.25 mm was used for PB and 0.2 mm for Ti-6-Al-4V, and thicknesses of 0.2, 0.25, and 0.3 mm were used for SS 304 Ni and SS 316. All materials were purchased from a company (KOS, Korea) specializing in metal wire.

### 2.2. Experimental Methods

#### 2.2.1. Biocompatibility

MTT (3-(4, 5-dimethyl-2-thiazolyl)-2, 5-diphenyl-2H-tetrazolium bromide) assay and cytotoxicity testing were performed in order to assess the biocompatibility of the experimental material. The procedure for each experiment conducted was as follows.

**(A) MTT Assay**

**(a) Assay Standards and Methods**

The cytotoxicity test of ISO 10993-5 for assessment of the cytotoxic potential of a test element (medical device) after direct contact was used as the assay standard.

The procedure composed with cell seeding, contact of the test element, incubation for more than 24 hours, preparation of the coloring solution of revelation, revelation of cytotoxicity, reading.

**(b) Extraction Condition**

The wires were segmented into 10 mm sections, and 40 sections were added to 3 mL of Dulbecco's Modified Eagle's Medium: nutrient mixture F-12 (DMEM/F-12, GIBCO). Extraction was performed at 37°C for 48 hours. The assignment of experimental and control groups was as follows (Table 1).

**(c) Experimental Method and Evaluation Standards**

**(1) Experimental Methods**

Mouse osteoblast (MC3T3 cell) was cultured for 24 hours with DMEM/F-12 (5% FBS, penicillin-streptomycin added). It was inspected for contamination before use. Media of sufficiently grown cells were removed, and media extracts of the experimental group and the control group were cultured separately for 24 hours. The media were then added to a plate with formazan crystal. After the formazan had dissolved, a Dymatech MRX ELISA microplate reader (Dynatech laboratories, Chantilly, VA, USA) was used to measure the absorbance at 540 nm. The average of 3 measurements was used to calculate the percentage of cell solubility. The result of the control group was used as the negative control.

**(B) Stain Test**

**(a) Experimental Method and Evaluation Standard**

The conditions for extraction and cell culture were performed as in an MTT assay. The cultured cells were stained with 0.3% crystal violet, and a stereoscopic microscope (Leica microsystem DE/EZ4) was used to compare their viability.

#### 2.2.2. Corrosion Stability

**(A) Corrosion Condition**

We prepared 5 cm of wire for each type. In order to consider body fluid conditions, 1 cm of each wire was dipped separately into 50 mL of Hank's solution (Table 2), which is a simulated body fluid, and current was applied at 0.5 mA for 60 minutes as a continuous wave, step response, 1 ms duration, and single-phase current. Current was supplied by a S88 stimulator (GRASS; USA: 0.01~0.5 mA).

**(B) Measurement of Corrosion**

A stereoscopic microscope (SMZ 1500, Nikon, Japan) and scanning electron microscope (SEM) were used to observe corrosion. The stereoscopic microscope was used to observe changes in color and shape, and SEM was used to observe surface changes due to corrosion.

### 2.3. Evaluation of Tensile Strength and Straightness

Wires selected for corrosion stability and biocompatibility were evaluated for travel speed and straightness with respect to thermal treatment conditions, and tensile strength was calculated with respect to the evaluated straight line. For the evaluation of straightness, the gap length was defined as the deviation from a reference line at a distance of 100 mm, to measure the extent of bending. The tensile strength was calculated by dividing the maximum tensile load before material breakdown by that of an original cross-sectional area of the sample.

## 3. Results

### 3.1. Properties of Electroacupuncture Needle Materials

Ti-6Al-4V is a Ti alloy with excellent biocompatibility that is used as a material for dental implants. Its electrical conductivity, however, is low, at 1.01% (IACS—International Annealed Copper Standard), and it is also expensive. SS 304 Ni is an existing SS 304 wire that is coated with Ni, whose high electrical conductivity, at 25% (IACS), improves the wire's conductivity. It is expected that this higher electrical conductivity can improve the effectiveness of electro acupuncture. SS 316 is the most commonly used stainless steel, along with SS 314. Compared to SS 314, SS 316 has a lower Cr- and a higher Ni-content, and Mo is added to it. It has a higher resistance to corrosion and creep but has inferior electrical conductivity, at 2.5% (IACS), as compared to 3.0% for SS 304 (IACS) (Table 3).

### 3.2. Biological Safety

#### 3.2.1. Corrosion Stability of Electroacupuncture Needle Material

**Figure 1:**
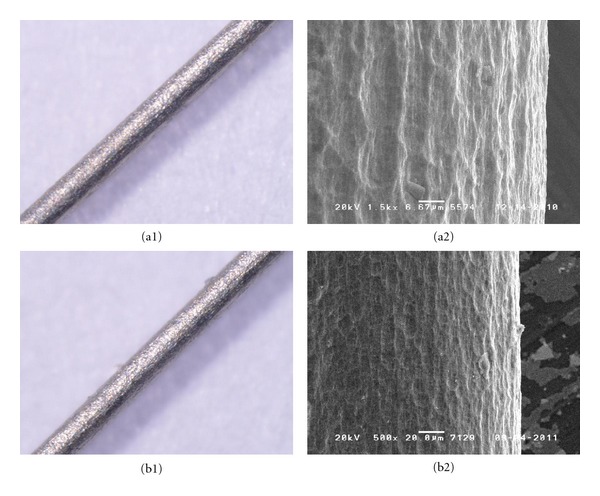
Corrosion test of Ti-6Al-4V: no corrosion was identified on the thickness of 0.20 mm (B is the control).

**Figure 2:**
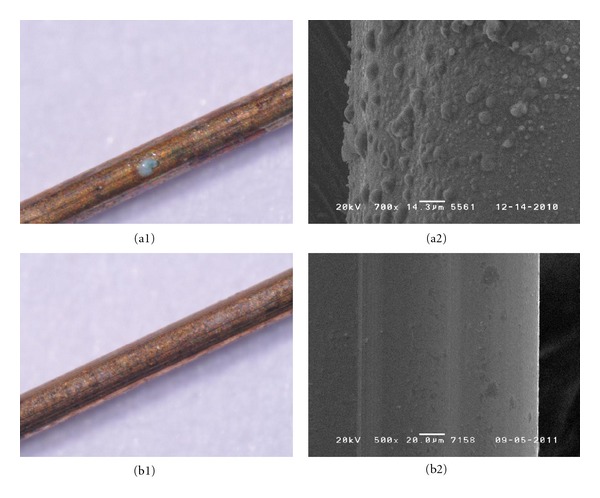
Corrosion test of PB: corrosion was identified on the thickness of 0.25 mm (B is the control).

**Figure 3:**
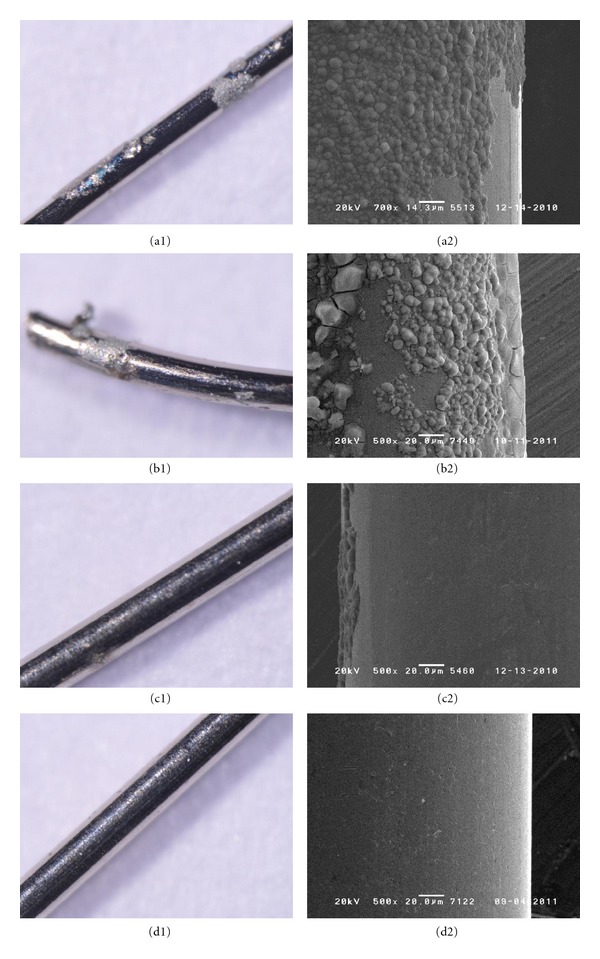
Corrosion test of STS 304 Ni: corrosion was identified on all thicknesses (A: 0.20 mm, B: 0.25 mm, C: 0.30 mm, D: control).

**Figure 4:**
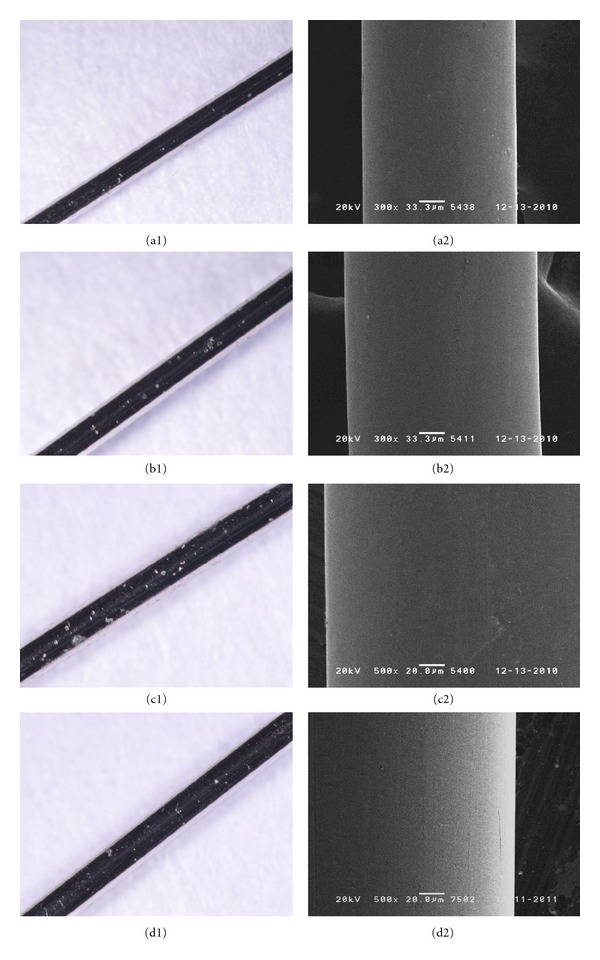
Corrosion test of STS 316: no corrosion was identified on any of the thicknesses (A: 0.20 mm, B: 0.25 mm, C: 0.30 mm, D: control).

The results of the corrosion stability tests of new acupuncture needle materials in simulated body fluid are as follows (Table 4). Examination with a stereoscopic microscope showed that there was no difference before and after electrical stimulus in Ti alloy (Figure 1). For PB, a stereoscopic microscope showed discoloring and SEM showed corrosion (Figure 2). SS 304 Ni showed corrosion with both a stereoscopic microscope and SEM, for all thicknesses (Figure 3). SS 316 showed a corrosion-like appearance under a stereoscopic microscope, but no corrosion using SEM (Figure 4 and Table 4).

#### 3.2.2. MTT Assay

**(A) 5.1 Microplate Reader Absorbance Analysis (540 *μ*m)**

**Figure 5:**
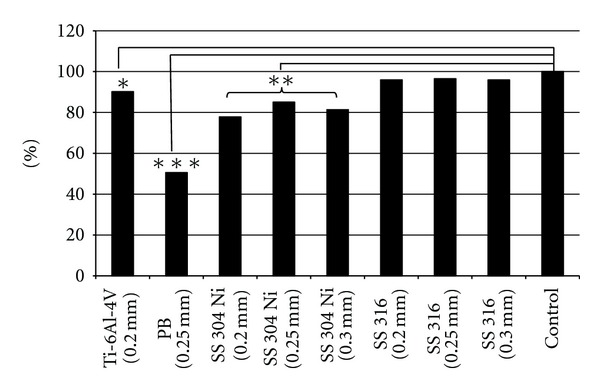
MTT assay result (*: *P* < 0.05, **: *P* < 0.01, ***: *P* < 0.001 versus control group).

When cell viability was expressed as a percentage with respect to the negative control, PB showed the lowest viability (50.5%) and SS 316 (0.25 mm) showed the highest viability (Table 5), (Figure 5).

Because there is no separate standard of cell viability for oriental medicine equipment, the standards of the Federation Dentaire Internationale (FDI) were used for our evaluation (Tables 6 and 7). In our results, Ti-6Al-4V, SS 316, and SS 304 Ni (0.25 mm and 0.3 mm) showed mild cytotoxicity, while SS 304 Ni 0.2 mm and phosphor bronze showed moderate cytotoxicity.

#### 3.2.3. Stain Test Results

**Figure 6:**
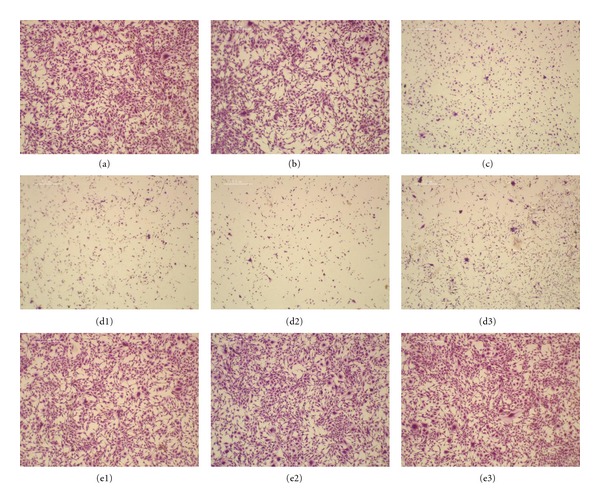
Stain test of each material. B (Ti-6Al-4V) and E (SS 316) show higher rate of survival, while (c) (PB) and (d) (SS 304 Ni) show a lower rate ((a) is the control).

In the MTT assay, the degree of cell viability was high, except for PB and SS 304 Ni 0.2 mm, but in the cell stain testing, PB and SS 314 Ni for all thicknesses showed low cell viability compared to the control group (Figure 6(a)), except for Ti-6Al-4V (Figure 6(b)) and SS 316 (Figure 6(e1), 6(e2), 6(e3)).

### 3.3. Straightness and Tensile Strength

In our evaluation of corrosion, only Ti-6Al-4V and SS 316 were not corroded. In the MTT assay, Ti-6Al-4V, SS 316, and SS 314 Ni showed excellent cell viability of grade 1 or higher. However, only Ti-6Al-4V and SS 316 showed moderate cell viability in the stain test. In terms of cost, Ti alloy (1720 KRW/kg) is about 70 times more expensive than SS 316 (24.5 KRW/kg) (Table 8). In terms of electrical conductivity, the conductivity of Ti-6Al-4V with 1.01% (IACS) was about 2.5 times lower than that of SS 316 with 2.5% (IACS). After consideration of the above results, SS 316 was selected as a candidate material for an electro acupuncture needle. After transforming the material into a needle shape, straightness and tensile strength tests were performed to check its conformity to standard specifications.

**(1) Manufacture of Straight Wire**

As a result of the linearization of SS 316 wire, wires with thicknesses of 0.20, 0.25, and 0.30 mm were all appropriate at a temperature condition of 700°C ± 3°C. The most appropriate straight product under 2.0 mm was obtained at a travel speed of 0.58 m/s ± 0.01 m/s (Table 9).

**(2) Evaluation of Straight Wire Tensile Strength**

**Figure 7:**
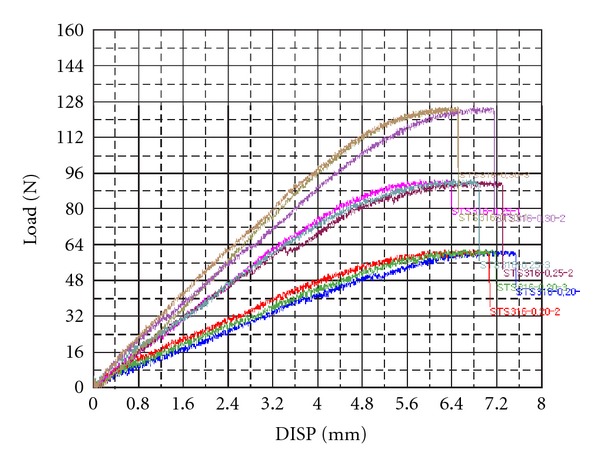
Tensile graph of SS 316(0.20, 0.25, 0.30 mm); result of tensile strength was 183~210 kgf/mm2, which is superior to the range of tensile strength of currently used SS 304.

Measurements of the tensile strength of the qualified wire showed that the range of tensile strength was 183~210 kgf/mm^2^, which is superior to the range of tensile strength of currently used SS 304, which is 170~190 kgf/mm^2^ (Table 10, Figure 7).

## 4. Discussion

 Electro acupuncture is a technique that applies an electrical stimulus to an inserted needle, and it is currently applied to a variety of illnesses in clinics worldwide [13]. However, the needles used in electro acupuncture, which correspond to electrodes for the low-frequency stimulation of meridian acupuncture points, are the same disposable needles that are used in conventional acupuncture. This has created a controversy regarding the corrosion of the needle in the course of the electrical stimulus treatment [5–7]. In order to resolve this controversy by discovering a new material that can replace the existing electrode and satisfy conditions for a disposable needle, for this study we selected 2 types of wires that have excellent electrical conductivity and 2 types of wires with a high degree of stability, both of which were commercially available. Phosphor bronze is a widely used contact terminal of the electronic device and known as a stable and a good conductor material. However, biological safety has not been confirmed. For this reason, we choose the material as a lowest reference of candidate. SS304 is conventional acupuncture needle material used in EA also. Ni-coated SS 304 was evaluated as composite material combined high conductive material with a conventional EA needle material, for batter electrical conductivity. Ti-6Al-4V is the most widely used titanium alloy in medical implant and known very safe. For this reason, we choose the material as a highest reference candidate. SS 316 has also been evaluated as a good candidate for the reason wide use in medical implant also, and other various invasive device material. A biocompatibility study, economic analysis, and corrosion testing after the application of electrical current showed that PB was unusable due to the severe cytotoxicity it displayed and that SS 304 Ni was also unsuitable, as it showed low cell viability in stain testing and showed corrosion after the application of current. SS 316 and Ti alloy performed well in terms of cell viability and cytotoxicity and did not exhibit corrosion under 0.5 mA continuous wave, step response, 1 ms duration, and single-phase current for 60 minutes. The results of economic analysis and electrical conductivity testing showed that Ti-6Al-4V (1720 KRW/kg) is about 70 times more expensive than SS 316 (24.5 KRW/kg) and that the electrical conductivity of Ti-6Al-4V, at 1.01% (IACS), was about 2.5 times lower than that of SS 316, at 2.5% (IACS). In addition, even in biological safety SS 316 showed better results than Ti-6Al-4V. As a result, SS 316 was selected as a candidate material for an electro acupuncture needle and was then tested for straightness and tensile strength in order to confirm its successful transformation into needle form and its conformity to standard—KS, JIS and GB (Korea Standard— KS, Japanese Industrial Standard—JIS, Guojia Biaozhun/National Standard/China—(GB) specifications. SS 316 satisfied straightness test under a condition of 0.58 m/s ± 0.01 m/s, and its range of tensile strength was 183~210 kgf/mm^2^, which is higher than the range of the currently used SS 304, which is 170~190 kgf/mm^2^. Based upon these overall results, it was confirmed that SS 316 is appropriate for use as a material for an acupuncture needle. This result is further supported by a report from Tang et al. [14] which states that SS 316 demonstrates superior resistance to electrochemical corrosion compared to SS 304 in both body fluid and cell growth environments. Clinical conditions, however, are much more complex than this, and a simple corrosion test in body fluid does not constitute a guarantee of safety. Therefore, wide-ranging research on the safety of an SS 316 needle with applied current under various conditions must be performed, and, based upon such researches, guidelines for safe usage should be developed for different treatment conditions. 

## 5. Conclusion

In order to develop new material for an acupuncture needle that is safe for electrical current stimulus, 4 types of commercially available materials were tested for their biological safety and risk of corrosion caused by applied current. Based upon our results, the following conclusions were reached.

1. SS 316 showed best biological safety and cost effectiveness as an electro acupuncture needle material.
2. Testing for straightness and tensile strength of SS 316 showed that it is suitable as an acupuncture needle under the condition of 0.58 m/s ± 0.01 m/s.

In summary, it was confirmed that a disposable needle capable of transmitting electrical stimulus can be manufactured using SS 316. If an animal study using an SS 316 needle is performed in the future to study the degree of corrosion under various electrical stimulus conditions and to research the materials capacity to provide safe treatment, this will facilitate the development of safer and more effective acupuncture treatment.

## Figures and Tables

**Table 1:** Allocation of experimental group and control group.

No.	Wire	Assignment
(1)	Ti-6-Al-4V 0.20 mm	Experimental group
(2)	Phosphor bronze 0.25 mm	Experimental group
(3)	SS 304 Ni 20 mm	Experimental group
(4)	SS 304 Ni 25 mm	Experimental group
(5)	SS 304 Ni 30 mm	Experimental group
(6)	SS 316 0.20	Experimental group
(7)	SS 316 0.25	Experimental group
(8)	SS 316 0.30	Experimental group
(9)	DMEM/F-12	Control group

**Table 2:** Chemical composition of Hank's solution.

Component	Concentration (mol dm^−3^)
NaCl	137.0
KCl	5.4
Na_2_HPO_4_	0.25
KH_2_PO_4_	0.44
CaCl_2_	1.3
MgSO_4_	1.0
NaHCO_3_	4.2

**Table 3:** Comparison of cost, conductivity, and tensile strength of wire material (1st of July, 2010).

Material	Diameter (mm)	Material	Tensile strength (N/mm^2^)	Electrical conductivity (% IACS)	Unit cost (1000 won/Kg)
Ti-6Al-4V	0.25	C 0.08%, Al 5.5~6.5%, Ni 0.05%, O 0.13%, Ti 88~90.08%, V 3.5~4.5%, Fe 0.25%, H 0.013%	2005	1.01	1720
Phosphor bronze	0.25	P 0.03–0.35%, Sn 4.5–9.0%	2131	15	16
SS 304 Ni	0.2	C 0.075%, Si 0.45%, Mn 1.25%, P 0.004%, Ni 8.47%	1685	25	22.5
SS 316	0.25	C below 0.08%, Si below 1.0%, Mn below 2.0%, Cr 16~18%, Ni 10~14%, Mo 2.0~3%	999	2.5	24.5

**Table 4:** Corrosion assessment of wire material in Hank's solution (x: corrosion was not observed. o: corrosion was observed).

Diameter (mm)	Ti-6Al-4V	Phosphor bronze	SS 304 Ni	SS 316
0.2	x	—	o	x
0.25	—	o	o	x
0.3	—	—	o	x

**Table 5:** The absorbance (540 nm) from MTT assay and viability.

	Absorbance					
	1st	2nd	3rd	Average	S.D.	Viability (average/control)
Ti-6Al-4V (0.20 mm)	0.337	0.34	0.336	0.338	0.002	*90.2%
PB (0.25 mm)	0.188	0.189	0.191	0.189	0.002	***50.58%
SS 304 Ni (0.20 mm)	0.289	0.295	0.29	0.291	0.003	**77.83%
SS 304 Ni (0.25 mm)	0.321	0.315	0.319	0.318	0.003	**85.049%
SS 304 Ni (0.30 mm)	0.304	0.306	0.304	0.305	0.001	**81.39%
SS 316 (0.20 mm)	0.362	0.361	0.355	0.36	0.004	95.99%
SS 316 (0.25 mm)	0.36	0.359	0.365	0.362	0.003	96.52%
SS 316 (0.30 mm)	0.358	0.357	0.362	0.359	0.003	95.9%
Control	0.376	0.374	0.373	0.374	0.002	100%

(*P < 0.05, **P < 0.01, ***P < 0.001 versus control).

**Table 6:** Definition of index values.

0	No observable lysis
1	Up to 20 percent
2	20–40 percent
3	40–60 percent
4	60–80 percent
5	Over 80 percent

**Table 7:** Response index and cytotoxicity.

Response index	Cytotoxicity
0	None
1	Mild
2-3	Moderate
4-5	Severe

**Table 8:** Results of safety and economic evaluation according to the material (x: corrosion was not observed. o: corrosion was observed).

Material	Thickness	Corrosion	Viability (MTT assay)	Viability (stain test)	Cost effectiveness
Ti-6Al-4V	0.20 mm	x	90.20%	High	Low
PB	0.25 mm	o	50.58%	Low	High
Ni co	0.20 mm	o	77.83%	Low	Normal
Ni co	0.25 mm	o	85.04%	Low	Normal
Ni co	0.30 mm	o	81.39%	Low	Normal
SS 316	0.20 mm	x	95.99%	High	Normal
SS 316	0.25 mm	x	96.52%	High	Normal
SS 316	0.30 mm	x	95.90%	High	Normal

**Table 9:** Process in accordance with the conditions of the wire straightness evaluation.

Classification	Temperature (°C)	Travel speed (m/s)	Result (mm)	Note
0.20	670	0.50	2.2	Fail
0.20	670	0.58	2.4	Fail
0.20	670	0.66	2.5	Fail
0.20	700	0.50	1.2	Pass
0.20	700	0.58	0.8	Pass
0.20	700	0.66	1.0	Pass
0.20	720	0.50	2.6	Fail
0.20	720	0.58	2.4	Fail
0.20	720	0.66	2.3	Fail
0.25	670	0.50	2.0	Pass
0.25	670	0.58	2.1	Fail
0.25	670	0.66	2.3	Fail
0.25	700	0.50	1.2	Pass
0.25	700	0.58	1.0	Pass
0.25	700	0.66	1.2	Pass
0.25	720	0.50	2.0	Pass
0.25	720	0.58	1.9	Pass
0.25	720	0.66	1.6	Pass
0.30	670	0.50	2.1	Fail
0.30	670	0.58	2.3	Fail
0.30	670	0.66	2.4	Fail
0.30	700	0.50	1.7	Pass
0.30	700	0.58	1.4	Pass
0.30	700	0.66	1.5	Pass
0.30	720	0.50	2.5	Fail
0.30	720	0.58	2.2	Fail
0.30	720	0.66	2.1	Fail

**Table 10:** Result of tensile test.

No.	Sample size	Sectional area	Maximum load	Tensile strength	Maximum displacement	Required time
SS 316-0.20-1	0.1997	0.03	6.28	209.333	7.540	00:00:45
SS 316-0.20-2	0.1997	0.03	6.32	210.667	7.070	00:00:42
SS 316-0.20-3	0.1997	0.03	6.32	210.657	7.200	00:00:43

SS 316-0.25-1	0.248	0.05	9.54	190.880	6.390	00:00:38
SS 316-0.25-2	0.248	0.05	9.46	189.200	7.310	00:00:43
SS 316-0.25-3	0.248	0.05	9.56	191.200	6.890	00:00:41

SS 316-0.30-1	0.297	0.07	12.86	183.714	6.510	00:00:39
SS 316-0.30-2	0.297	0.07	12.86	183.714	7.170	00:00:43
SS 316-0.30-3	0.297	0.07	12.90	184.285	6.530	00:00:39
